# A New Society

**Published:** 1865

**Authors:** 


### A NEW SOCIETY.

The junior members of the profession in our city have organized a society, called "THE DENTAL COLLEGE SOCIETY OF CINCINNATI."

It is composed of those who are or have been students in the College. There are twelve or fifteen members. It is organized with special reference to the students during the sessions of the Institution. This society will doubtless result in great good to its members.

The officers are:

WM. TAFT, *President.*

H. BARTLESON, *Vice-President.*

JAS. J. TAYLOR, *Recording Secretary.*

J. P. COLLINS, *Corresponding Secretary.*

WM. PABODIE, *Treasurer.*

Meetings held semi-monthly.

